# Dietary Effects on Microbiota—New Trends with Gluten-Free or Paleo Diet

**DOI:** 10.3390/medsci6040092

**Published:** 2018-10-18

**Authors:** Yurdagül Zopf, Dejan Reljic, Walburga Dieterich

**Affiliations:** 1 Medical Clinic 1, Friedrich-Alexander-Universität Erlangen-Nürnberg, Ulmenweg 18, 91054 Erlangen, Germany; 2 Hector Center of Excellence for Nutrition, Exercise and Sports, University of Erlangen-Nürnberg, 91054 Erlangen, Germany; dejan.reljic@uk-erlangen.de (D.R.); walburga.dieterich@uk-erlangen.de (W.D.)

**Keywords:** diet, gluten-free, microbiota, Paleo diet

## Abstract

A well-balanced diet is the basis for a healthy life. Both the western diet and special diets can have a relevant impact on the microbiome and promote the development of various diseases. There has been an increase in food-related disorders in recent years, largely associated with dramatic changes in food consumption trends and main nutrients. A major response to food intolerances has been the adoption of new dietary trends involving the reduction or exclusion of specific food ingredients. Especially gluten-containing, but also gluten-free cereals are in the cross-fire. Supporters of the gluten-free diet argue that gluten triggers inflammation and related diseases, while followers of the Paleo diet drastically impeach all cereals as dangerous for human health. To date, no controlled studies support or reject a positive health effect of a gluten-free or cereal-free diet. Future large-scale studies need to evaluate the effect of gluten-containing and gluten-free cereals and the various diets on human health, inflammatory parameters, clinical symptoms, and the gut microbiota (including the bacteria, fungi, and viruses). Dietary-associated changes in compositional and functional microbiota traits should be correlated with the health status for the future development of dietary recommendations and potential clinical interventions.

## 1. Introduction

A healthy and well-balanced diet is the basis for a healthy life and mental well-being. In the past 50–100 years, Westernized communities have seen dramatic changes in the physiological and psychological relationship with food, which have challenged our long-term adaptation to nutrients. On the one hand, the intake of finished products strongly increased, together with the consumption of novel chemical compounds present in the nutritional formulation. For example, a variety of additives, including preservative agents, emulsifiers, colorants, and flavor enhancers, are now commonly used in the food industry and sourdough is mainly replaced by yeast fermentation. On the other hand, novel forms of nutrition, including vegetarian and vegan diets, low-carb and low-fat diets, and a gluten-free diet (GFD) or cereal-free Paleo diet, have gained popularity. While these “elimination diets” are assumed to be healthier, up to date there are no randomized and controlled studies which confirm their constitutional effect for healthy individuals. In contrast, several studies have shown that healthy people who consume elimination diets typically face an increased risk of malnutrition, including deficiencies in minerals and vitamins [1,2].

The surge of elimination diets has been mostly justified by mild or severe clinical intolerances to common nutritional ingredients. However, most of the claimed intolerances are not clinically verified. The spectrum of nutrients known to trigger intolerances and their related symptoms is wide: sugar can cause gastrointestinal complaints in affected individuals [3], gluten is the etiological and damaging agent in patients with celiac disease [4], and food proteins, e.g., nuts, soya, fish, eggs, milk, or wheat proteins may provoke allergic responses which, at their most severe, can be life-threatening [5,6]. In other diseases, such as irritable bowel syndrome (IBS) and psoriasis, typically no dietary trigger is known; nevertheless, patients often show improvement in clinical symptoms after dietary adjustment, such as the elimination of distinct nutrient components (e.g., carbohydrates in IBS or psoriasis, gluten in non-celiac gluten-sensitivity (NCGS)) [7,8,9].

In recent years, the importance of the intestinal microbiota and its interaction with the host has become evident. The intestinal immune system plays a central role in stabilizing the host defense by protecting against microorganisms. If this system collapses, there will be a risk of immune-mediated diseases, e.g., autoimmune reactions. The microbiota proved to be a very complex and well-balanced intestinal ecosystem that influences gut homeostasis and delivers valuable food metabolites, for instance, vitamins, or short-chain fatty acids, which are essential fuels for the host. Studies on mice have shown that gut microbes are responsible for an increased energy uptake through the fermentation of complex fibers that are otherwise indigestible for the host. It is speculated that in humans eating a Western diet nearly 10% of the energy uptake originates from short-chain fatty acids that are derived from fiber degradation by gut bacteria [10]. However, the gut microbiota has also gained much attention as a potential causative agent of several intestinal disorders associated to food intolerances [11]. A growing body of evidence indicates that dysbiosis of the gut microbiota, i.e., microbial quantitative imbalance or compositional alteration, is strongly associated with IBS, food intolerance, obesity, Crohn’s disease, and other intestinal inflammatory diseases [12,13,14].

Despite the wide differences in gut microbiota, human and mice harbor beneficial bacteria mainly belonging to phylum Firmicutes and Bacteroidetes. A dramatic reduction in Bacteroidetes and increase in Firmicutes has been observed in obese mice compared to lean siblings, and it is suggested that obesity alters the diversity of gut microbiota [15]. In accordance, obese humans have shown decreased proportions of Bacteroidetes accompanied by increased levels of Firmicutes, and low-calorie diets have caused a considerable increase in Bacteroidetes [16]. The exact mechanism through which microbiota influence or favor obesity is still unknown. Bäckhed et al. have stated that germ-free mice are protected from diet-induced obesity by mechanisms leading to increased fatty acid metabolism [17]. In addition, studies with obese and diabetic mice have revealed an increased intestinal permeability that can be reduced by enrichment of *Bifidobacterium* species (spp). The feeding of mice with prebiotic oligofructose causes a change in the gut microbiota and an increased expression of the proglucagon-derived peptide GLP-2 which results in lower intestinal permeability and improved epithelial integrity [18]. Recent data also shows that hyperglycemia influences intestinal epithelial permeability and may favor systemic infection and inflammation in obesity and diabetes [19]. Furthermore, gut microbiota has been discussed as playing a crucial part in the formation of intestinal capillary networks because they promote glycosylation of tissue factor (TF), a membrane receptor that is responsible for vascular remodeling [20,21].

A dysbiotic status can be driven by infections, antibiotics, genetics, and environmental changes, but also specific diets. While causation/correlation patterns in clinical results are not always clear, meaning that dysbiosis could be regarded as a symptom rather than a trigger, restoration of a balanced microbiota through fecal transplants was shown to cure 98% of patients affected by *Clostridium difficile* infections [22], and was also reported in a patient with refractory celiac disease [23]. This largely supports a potential causative role of the gut microbiota in other intestinal disorders.

**Figure 1:**
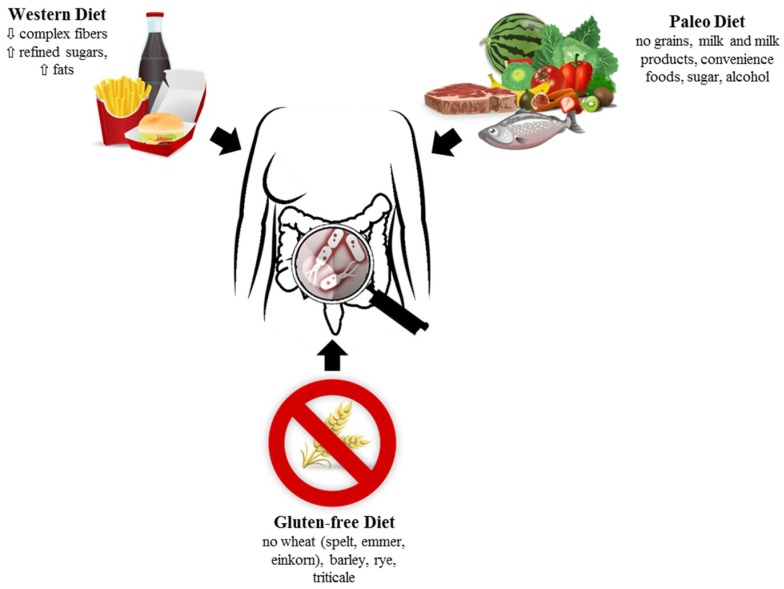
Diets strongly influence gut microbiota. Further studies must evaluate the impact of diets on gut health.

Several studies showed that changes in food intake significantly altered the gut microbiota. In fact, the availability of distinct food components favors the selective enrichment of microorganisms capable of exploiting these nutrients and supports microbial metabolic cross-feeding, leading to the maintenance of a diverse and balanced community [24]. Even short-term changes in diet were shown to significantly alter the gut microbiota structure [25] (Figure 1).

## 2. Western Diet—Whole Grain, Vegetarian, Fruit, Vegetables, and Nuts

The diet of people living in Western countries is rather low in fiber and provides a high amount of fat and refined carbohydrates compared with the diet of people in non-industrialized countries [26]. The different diets seem to affect the microbiome and the diversity of the intestinal microbiome significantly [27]. Also, the mere absence or presence of meat and products made therefrom can also significantly affect the microbiome. There are indications that omnivores accumulate *Clostridium* species, which are mainly butyrate-producing bacteria. In comparison, vegetarians tend to accumulate the groups of *Bacteroides* and *Prevotella* [28].

Diverse studies have investigated the influence of whole-wheat flakes on the composition of the gut microbiota [29]. For example, a controlled study showed that after three weeks of corn-based whole grain consumption, the *Bifidobacteria*, *Lactobacillus*, and *Enterococcus* were elevated in the subjects’ stool [30], but there was no effect on short-chain fatty acid (SCFA) levels. However, Kopf et al. could demonstrate a decrease of subclinical inflammation after the ingestion of whole grains [31].

Already minimal variations in dietary fiber composition promote environmental conditions for specialized microorganisms. Rat studies have displayed a relation between soluble arabinoxylan and *Akkermansia* and the production of propionic acid, whereas β-glycan or insoluble arabinoxylan favored butyrate producers, e.g., *Lactobacillus*, *Blautia*, and *Allobaculum* [32]. Even a single intake or consumption of rye kernel bread on three consecutive evenings has shown variations in gut microbiota in young healthy volunteers with a higher abundance of *Faecalibacterium* and *Prevotella* but a reduced proportion of *Bacteroides* spp. in comparison to white wheat bread intake. A positive association of *Prevotella* with plasma levels of the brain-derived neurotrophic factor is noticed, but further studies are necessary to explore a possible protective effect of *Prevotella* on neurodegenerative processes [33].

In several studies, the influence of fruit on the composition of the intestinal microbiota was investigated. Vendrame et al., for instance, studied the influence of a wild blueberry drink in a placebo-controlled study and found an increased amount of *Bifidobacterium* spp. in the stool of volunteers [34]. Anhe et al., showed a decreased metabolic inflammation and endotoxaemia after treatment with camu camu (*Myrciaria dubia*), so that the antioxidative properties of fruit may unfold with the change in the microbiota [35].

With regard to nuts, the influence of almonds and pistachios was investigated on the composition of the human intestinal microbiota in randomized controlled trials [36]. The pistachios seem to have a greater influence on the microbiota than almonds with a slightly higher production of butyrate. Holscher et al., demonstrated that a walnut diet favors the increase of the relative abundance of butyrate-producing species belonging to Firmicutes, e.g., *Clostridium* clusters XIVa and IV. Therefore, the proposed health effect of walnuts may be partly due to the microbiota [37].

Only a few studies examined the influence between the consumption of vegetables and the composition of intestinal microbiota. After eating chickpeas, fewer subjects were positive for *Clostridium* clusters without any effect on SCFA concentrations [38]. Another study examined the influence of soymilk on the gut microbiota compared to cow milk. After the intervention period, the soymilk group showed a reduced Firmicutes-to-Bacteroidetes ratio compared to the baseline values in comparison to the bovine milk group [39]. In addition, Kopf et al. could demonstrate that a vegetable-enriched food can achieve an anti-inflammatory effect by altering microbial diversity [31].

Several studies examined the influence of inulin on human gut microbiota. For example, one study investigated the effect of a mixture of inulin and partially hydrolyzed guar gum (I-PHGG) or maltodextrin on intestinal microbiota. The total numbers of *Clostridium* spp. were reduced in the fiber group, but there were no differences in the concentrations of SCFA [40]. Another study showed a significant increase in *Bifidobacterium* after inulin administration compared to maltodextrin, even when no differences in SCFA concentrations were present [41]. On the other hand, Alexander et al. could present a modulation of gut microbiota and metabolites after inulin treatment with an increase of Firmicutes and a decrease of Proteobacteria [42]. Singh et al. showed metabolic effects after inulin treatment with decreased calorie intake and upregulated satiety hormones [43].

### 2.1. Fat

The more a diet is enriched by a particular macronutrient, the greater the shift in the levels of the other macronutrients is. For example, high-fat diets are usually low in carbohydrates and have a lack of complex carbohydrates. This shift in nutritive compounds, as well as the absorbed fat quantity and quality, can influence the composition of the intestinal microbiota in a highly relevant fashion. Preliminary data suggest that ingested fat levels modulate the community of intestinal microbiota via their effect on bile acid secretion and bile acid composition. Due to their selective antimicrobial activity, bile acids can significantly alter the intestinal microbiota under a high-fat diet [44].

So far, little is known about the quality of fat and its effect on bile acid composition and thus on the gut microbiota pattern. Wu et al. reported an altered intestinal microbiome depending on the intake of saturated fats with an increase in the *Bacteroides* enterotype but a reduction of the *Prevotella* enterotype [25]. In another intervention study, a high-fat animal-based diet resulted in increased levels of deoxycholic acid in stool and a significant change in the microbiota, leading to an increase in bile-acid-tolerant bacteria [45]. Just et al. were able to show that unfavorable fat intake and accumulation of bile acids can lead to a change in the microbiota and an increase in liver fat [46]. These results confirm a link between dietary fat, bile acid metabolism, and changes in intestinal microbiota.

### 2.2. Protein

The effect of protein on the human microbiota composition has been studied only to a limited extent. A high-protein and low-carbohydrate diet has been shown to affect gut microbiota in overweight men. After a four-week period, the high protein diet resulted in an increase in branched-chain fatty acids, a decrease in butyrate concentrations, and a decrease in *Roseburia* and *Eubacterium* counts [47]. In addition, Watanabe et al. were able to demonstrate that a soybean protein can trigger considerable changes in gut microbiota and significantly reduce the risk of weight gain and accumulation of adipose tissue [48]. Digested bioactive peptides from soybean are said to have a hypocholesterolemic property. It is suggested that this effect influences the low-density lipoprotein receptor or bile acid regulation, but an involvement of microbiota is also assumed. In this context, rats that were fed with 20% soybean protein showed bacterial shifts with increased *Enterococcus* but diminished proportions of *Ruminoccoccus* and *Lactobacilli*. Golden Syrian hamsters had reduced levels of Bacteroidetes but enriched Bifidobacteriaceae, Clostridiales, and Deferribacteriacea after three weeks supplementation with soy protein concentrates [49]. Although gut microbiota are suggested as being involved in cholesterol homeostasis, future studies must clarify to what extent microbial shifts are responsible in cholesterol metabolisms [50].

## 3. Gluten as Main Culprit—Celiac Disease

As mentioned above, elimination diets are necessary for some patients. Gluten, in particular, has become a main intolerance factor. For patients with celiac disease (CD), a strict lifelong gluten-free diet is mandatory and the only available therapy. In patients with a genetic predisposition (HLA DQ2 or DQ8), dietary gluten triggers the activation of gluten-specific T cells and results in the remodeling and destruction of the intestinal mucosa with crypt hyperplasia and villi atrophy [4]. In recent years, the prevalence of celiac disease has increased [51,52,53]. In part, this is caused by improved serological tests and the growing awareness of physicians.

The amino acid sequence of gluten is unique, with high amounts of proline and glutamine. This special primary structure is the reason why human gastric, pancreatic, or small intestinal proteinases cannot completely degrade gluten and up to 33-mer gluten fragments are found in the intestine. These long fragments displayed a potent T cell stimulation without the need of further processing by antigen-presenting cells [54]. The gluten challenge induced a mucosal cytokine response, increased intraepithelial lymphocytes, and interferon gamma (IFNγ) secretion in celiac patients [55]. Gluten peptides were also able to induce maturation and activation of dendritic cells in CD, with enhanced expression of surface maturation markers and increased secretion of chemokines and cytokines [56,57]. Most intriguingly, gluten also induced the maturation and activation of monocytes into dendritic cells in healthy individuals. However, dendritic cells of healthy controls showed less stimulatory potential, suggesting that healthy individuals are able to control and downregulate the gluten-induced activation of the immune system [58]. Together, these data clearly hint to a stimulatory potential of gluten that is tightly and efficiently controlled in healthy individuals but may be able to trigger or maintain a permanent immunological activation in responsive patients.

Interestingly, breeding and culture of more profitable wheat species, as well as variations in bread formulation, are thought to be responsible for the increasing incidence in CD. In this context, studies with ancient wheat species displayed less toxicity in in vitro assays [59], and the use of sourdough rigorously reduced gluten content in bakery products since sourdough microorganisms are able to degrade gluten [60]. The probiotic *Bifidobacterium lactis* is able to inhibit gluten-induced increased epithelial cell permeability of in vitro cultured colon carcinoma cell lines (CaCo2). However, it remains unclear, whether this is a direct effect of the *Bifidobacterium* on CaCo2 cells or caused by the degradation of gluten by bacterial proteases [61].

It was noticed that antibiotic use in childhood correlated with the onset of CD, and especially cephalosporin strongly increased the risk of CD. It is speculated that gastrointestinal infections and the use of antibiotics alter the gut microbiota, which may influence the development of CD [62]. Feces from newborns with a genetic risk for celiac disease showed reduced numbers of *Bifidobacterium* spp., especially *Bifidobacterium longum*, but a high abundance of *Bacillus fragilis* and *Staphlyococcus* spp., both belonging to Firmicutes. Interestingly, breastfeeding modified the microbiota pattern and resulted in a shift to beneficial bacteria with increased numbers of *Bifidobacterium* spp. but lower numbers of *B. fragilis* [63]. It is suggested that host genotype together with diet influence the gut colonization early in life. A dysbiosis was also demonstrated in children with active CD. These patients showed increased Gram-negative bacteria, *Bacteroides*, and *Escherichia coli* that were normalized under a gluten-free diet [63].

Furthermore, the intestinal microbiota seem to play a crucial role in the manifestation of CD. In this context, it was shown that adult patients with CD and persisting gastrointestinal symptoms or anemia possess a reduced microbial diversity, which is dominated by Proteobacteria [64]. Another study confirmed this enrichment of Proteobacteria, and displayed reduced numbers of Firmicutes and Actinobacteria. *Neisseria flavescens* was the most abundant *Neisseria* species in duodenum samples of patients with active CD and was able to induce an inflammatory response in dendritic cells and ex vivo mucosal samples. The dysbiosis and increase in microbiota with inflammatory potential are suggested to contribute to the maintenance of clinical symptoms in celiac disease [65].

Remarkably, several studies demonstrated that a gluten-free diet also caused a reduction in the proportion of *Bifidobacterium* spp., combined with an increase in *Enterobacteriacea* and *Escherichia coli* [66,67]. The loss in beneficial bacteria under a gluten-free diet is the basis for investigating the supplementation of celiac patients with probiotics in order to support the recovery and maintenance of healthy gut microbiota. Indeed, promising results derived from a double-blind placebo-controlled study. When children with active CD received a capsule containing *Bifidobacterium longum* CECT 7347 in addition to a gluten-free diet, this resulted in an improved health status compared to children that received the placebo compound [68]. Furthermore, the supplementation with *Bifidobacterium infantis* alleviated gastrointestinal symptoms in newly diagnosed adult patients with CD, but the probiotic was not able to affect the abnormal intestinal permeability in these patients. Further studies must prove the beneficial effect of probiotics in the recovery of gut microbiota in CD and on the gut mucosal barrier [69].

## 4. Gluten—Bad Guy in Non-Celiac Gluten-Sensitivity (NCGS)

Non-celiac gluten-sensitivity is now considered as a separate clinical entity. Although the clinical symptoms resemble the complaints in CD and strongly correlate with the intake of gluten, NCGS is clearly distinguishable from CD or wheat allergy. To date, no disease-specific serological parameters are known and only a moderate stimulation of the intestinal immune system with elevated intraepithelial cells was described. Currently, a GFD is the only adequate therapy, although some studies have cast doubt on gluten as the main or sole culprit in NCGS [70,71]. Other cereal components, like the polyfructose inulin or arabinoxylan-oligosaccharides, may be involved in the disease manifestation. In this context, a diet with low amounts of fructose and di- and oligosaccharides (FODMAP) resulted in significant improvement of gastrointestinal symptoms in most patients with NCGS [70,72].

In addition, amylase trypsin inhibitors (ATI) that are found in high amounts in cereals and are very stable to digestion or cooking, received much attention because of their ability to activate the naive immune system and maintain inflammatory processes [73]. ATIs from modern gluten-containing cereals (wheat, rye, and barley) display a high inflammatory potential in biological assays whereas ATIs from ancient wheat cultivars (emmer, einkorn, and spelt) have less bioactivity [74]. Recent data in humanized mice underline the importance of ATIs as activators of the innate immune system, causing increased T cell proliferation and cytokine production, and indicate ATIs as adjuvants of allergy [75]. However, the participation of ATI in pathogenesis of NCGS is still to be proved.

Our own data has shown that NCGS patients profited with partial symptom improvement while consuming a low FODMAP diet and completely normalized under GFD in regard to all clinical symptoms, stool consistency, and psychological well-being [76]. Most intriguingly was the reduction of intestinal intraepithelial lymphocytes under GFD, thus underlining the involvement of the immune system and the positive influence of the diets on the ongoing immune stimulation in NCGS. Significant alterations in microbial patterns were noticed already after two-week dietary modifications, although the individual bacterial enterotype was highly stable. The low FODMAP diet resulted in reduced *Lachnospiraceae* and *Bifidobacteriaceae*, and the GFD diet caused an increase in *Bacteroidaceae* in NCGS patients, thus adapting to the values of healthy controls. There were significant differences in bacterial families between NCGS patients and healthy controls. Genus analysis revealed much more significant variations caused by diets in NCGS than healthy controls. As expected, the changes in intestinal bacteria also showed an effect on metabolism concerning the ability of dehalogenation, ammonia oxidizing, sulfate reducing, and xylan and cellulose degradation, especially under GFD. Hence, a variation in diet had a curative effect on clinical symptoms and well-being in NCGS and displayed a huge impact on microbial pattern and metabolisms. Although a GFD resolves the clinical symptoms in NCGS, the role of gluten is still unclear and provokes further research. A multi-stage process with the involvement of carbohydrates and gluten in the course of NCGS is suggested [76].

## 5. Paleo Diet

New dietary trends include the Paleo diet, which is free of all cereals. Supporters of this diet argue that especially gluten but also gluten-free cereals were absent in the ancient hunters and gatherers’ diet. It is thought that the inclusion of cereals in the human diet started with the rise of agriculture, about 10,000 years ago, but that there has been no adequate adaptation of the human gut or microbiota to this drastic dietary change, although this argument is still controversially debated. Recent fecal metabolome analysis of Hadza hunter-gatherers from Tanzania, which should mirror our ancient gut microbiota before the inclusion of gluten, has clearly shown the main differences in microbial pattern and intestinal metabolites compared to urban-living Italians. Stool samples from Hadza hunters have been collected during the rainy season, when the dominant food is plant-based (with tubers, baobab, and honey), and game meat is rare. Most of the tubers contain a high moisture content and indigestible fibers, which are expectorated during chewing, thus the Hadza diet is enriched in monosaccharides, starch, and protein, but low in fat. The nutrition of the Italian comparison group has been adapted to the Mediterranean diet, with plenty of plant foods, fresh fruit, pasta, bread, and olive oil and moderate amounts of dairy and meat. Only a minor proportion of carbohydrates is derived from fibers [77].

The gut microbiota from Hadza show a higher microbial richness and biodiversity compared to microbiota from Italian controls. The Hadza microbiota is dominated by phylum Firmicutes (72%), Bacteroidetes (17%), Proteobacteria (6%), and Spirochaetes (3%). The high proportions of Proteobacteria and Spirochaetes in Hadza feces, especially, are considerably distinct from very low levels of these phyla in the Italian control group. The determination of the genus level reveals increased quantities of *Prevotella* (Bacteroidetes), *Treponema* (Spirochaetes), and unclassified Bacteroidetes in Hadza feces, but an absence of *Bifidobacteria*. The genus *Prevotella* and *Treponema* possess the ability for xylan degradation, and the phylum Firmicutes also harbors several fiber-degrading species. Thus, it is suggested that these microbiota are the prerequisite for proper digestion of the glycan and fiber-rich diet consumed by Hadza [77].

Interestingly, Hadza feces show an unusual pattern of Clostridiales with a reduction of butyrate-producing *Clostridium* clusters IV and XIV, which are considered to be beneficial bacteria, and Hadza microbiota also possess distinct enrichment of opportunistic bacteria, e.g., Proteobacteria, or *Treponema*. In addition, the absence of *Bifidobacteria* in Hadza gut microbiota is of special interest because these bacteria are found in 1–10% of Western adults’ gut microbiota and *Bifidobacteria* are considered as valuable probiotic agents for gastrointestinal disorders [78].

When investigating metabolic activity, there is striking evidence for the increased concentration of hexoses in Hadza feces compared to the Italian control samples (50.5% vs. 16.3%). The hexoses may be enriched because the Hadza diet is dominated by indigestible polysaccharides and fibers that pass through the small intestine, reach the colon, and are processed by local microorganisms. There is also a surplus of sphingolipids and glycerophosholipids but a strong depletion of amino acids and biogenic amines in the Hadza metabolome. The original source for sphingolipids and glycerophospholipids in feces is not known, because sphingo- and phospholipids are found in phylum Bacteroidetes but are also naturally present in host membranes. The metabolism of sphingolipids is further influenced by bile degradation, and since a typical high-fiber diet from Hadza hunters reduces the excretion of bile acid, this may promote higher concentrations of sphingolipids. Independent of the origin, sphingolipids and glycerophosholipids have been reported to exert an anti-inflammatory effect and may be responsible for the low immune stress in Hadza hunters [79]. Interestingly, current studies have shown a high structural similarity between bacterial- and mammalian-derived sphingolipids and a role of bacterial sphingolipids in the maturation of immune system has been described [80].

In summary, the remarkable microbial community with an altered metabolome from Hadza feces may be an adaptation and precondition for the specialized form of nutrition and may yield a benefit for this atypical lifestyle. However, further studies are necessary to clarify the impact of a Paleo diet on gut microbiota and health outcomes in the context of Western genetic and environmental conditions.

## 6. Discussion

In the course of life, the microbiome changes constantly. Even before birth, the flora of the mother may affect the microbial composition of the placenta. Afterwards, the type of delivery (vaginal or caesarean section), the influence of diet as a baby (breast milk vs. milk replacement products), genetics, age, educational status, health status, and diet in adulthood influence the microbial composition. At an advanced age, decreasing physical activity, altered eating habits, inflammatory conditions, and taking medications can alter the intestinal flora [13,81,82,83,84].

Nutrition has a very special influence on the microbiome as it is an important factor throughout age. Food components that are indigestible to human enzymes (e.g., fibers) provide substrates for microbial metabolism in the gut. Because bacteria are specialized in the fermentation of various substrates, complex diets can lead to a number of metabolic products, especially vitamins and SCFAs, which are vital to human health [85].

Information on the composition of the colon microbiota comes mainly from the analysis of stool samples. Based on molecular analysis, most bacteria belong to the phyla Bacteroidetes and Firmicutes [86]. The Gram-negative Bacteroidetes include the genera *Bacteroides* and *Prevotella*. These organisms have the ability to utilize a wide variety of substrates and are major producers of propionate [87]. Firmicutes include several species identified as dominant butyrate producers [88] and specialized degradants of indigestible polysaccharides [89].

Recently, it has been suggested that cereals and especially glutens are responsible for triggering the pathophysiology of many illnesses, including autoimmune diseases. This is the reason why the consumption of gluten-free products gains great popularity and the trade of gluten-free products achieves a big economical market. In view of the inflammatory capacity of gluten, one cannot completely rule out a fundamental effect of cereals on gut health or microbiota composition. However, conflicting results have come from fecal analysis that reveal that consuming a GFD results in a decrease of beneficial and increase of unhealthy gut bacteria in healthy individuals, thus supporting a positive effect of gluten [90]. The cereal-free Paleo diet reveals major differences in microbiota and metabolome and challenges our understanding of beneficial gut microbiota [77]. However, more studies are needed to clarify the impact of a Paleo diet on microbiota and gut health. In conclusion, the next-generation sequencing technology yields huge amounts of data and revolutionizes our understanding of the gut community. Besides bacteria, other microorganisms, e.g., viruses, bacteriophages, or fungi, are of great importance. Since microbiota are able to quickly adapt to changing conditions, in-depth knowledge of a microbial pattern will help to determine the influence of genetics or the environment. The identification of reliable, health- and disease-specific microbial indicator taxa will be a prerequisite for a balanced diet and to develop personalized dietary intervention protocols for patients according to their diseases. Thus, opening new therapeutic strategies. The identification of interactions among gut residents, their metabolic activity, and cross-feeding will be the most important and exciting task in this fascinating research area.
